# Disease Severity in Treatment Resistant Schizophrenia Patients Is Mainly Affected by Negative Symptoms, Which Mediate the Effects of Cognitive Dysfunctions and Neurological Soft Signs

**DOI:** 10.3389/fpsyt.2018.00553

**Published:** 2018-10-31

**Authors:** Felice Iasevoli, Camilla Avagliano, Benedetta Altavilla, Annarita Barone, Luigi D'Ambrosio, Marta Matrone, Danilo Notar Francesco, Eugenio Razzino, Andrea de Bartolomeis

**Affiliations:** Section of Psychiatry - Unit on Treatment Resistant Psychosis, Laboratory of Molecular and Translational Psychiatry, Department of Neuroscience, University School of Medicine Federico II, Naples, Italy

**Keywords:** psychosis, refractory, clozapine, antipsychotics, positive symptoms, response

## Abstract

This *post-hoc* study was aimed at assessing whether disease severity was higher in a sample of Treatment Resistant Schizophrenia patients (TRS) compared to schizophrenia patients responsive to antipsychotics (non-TRS). Determinants of disease severity were also investigated in these groups. Eligible patients were screened by standardized diagnostic algorithm to categorize them as TRS or non-TRS. All patients underwent the following assessments: CGI-S; PANSS; DAI; NES; a battery of cognitive tests. Socio-demographic and clinical variables were also recorded. TRS patients exhibited significantly higher disease severity and psychotic symptoms, either as PANSS total score or subscales' scores. A preliminary correlation analysis ruled out clinical and cognitive variables not associated with disease severity in the two groups. Hierarchical linear regression showed that negative symptoms were the clinical variable explaining the highest part of variation in disease severity in TRS, while in non-TRS patients PANSS-General Psychopathology was the variable explaining the highest variation. Mediation analysis showed that negative symptoms mediate the effects of verbal fluency dysfunctions and high-level neurological soft signs (NSS) on TRS' disease severity. These results show that determinants of disease severity sharply differ in TRS and non-TRS patients, and let hypothesize that TRS may stem from cognitive disfunctions and putatively neurodevelopmental aberrations.

## Introduction

Treatment Resistant Schizophrenia (TRS) is a major challenge in clinical management and therapy of schizophrenia (1), which *per se* is among the most relevant causes of morbidity worldwide (2). TRS is defined as the lack of response to a number of antipsychotic agents, which causes the patients to be actively symptomatic and to not gain symptom remission and functional recovery (3). Accordingly, TRS has been associated to more severe social disability (4), whose determinants appear to strongly diverge from that in responder schizophrenia patients (i.e., non-TRS) (5, 6). Also, TRS may represent a categorically distinct subtype of schizophrenia (7), as also suggested by clinical data showing higher severity of neurological soft signs (NSS) in these patients (8), a marker of aberrant brain development (9).

In this study, we evaluated whether disease severity differed in TRS vs. non-TRS patients. As a subsequent step, we tried to delineate the clinical factors influencing disease severity in these two groups.

## Methods

This *post-hoc* analysis used data from a previous cross-sectional naturalistic study (6). Patients' recruitment continued after the above-mentioned report, and therefore the present study includes data from an expanded sample compared to that earlier one.

Patients were referred to our academic Outpatient Unit on Treatment Resistant Psychosis, University “Federico II” of Naples, by community psychiatrists for evaluation of putative TRS, as they suffered from psychotic symptoms apparently non-responding to antipsychotic agents. All consecutive patients meeting criteria for eligibility were recruited.

Inclusion criteria were: (i) age within the 18–65-year range; (ii) diagnosis of schizophrenia; (iii) being treated with antipsychotics; (iv) stabilized symptoms, including persistent psychotic symptoms with no evidence of actual or recent (i.e., in the last 3 months prior assessments) worsening. Exclusion criteria were: (i) intellectual disability (according to DSM-5 diagnostic criteria); (ii) severe medical diseases; (iii) non-schizophrenia psychotic disorders; (iv) psychotic symptoms due to another medical condition or to substances/medications.

All patients signed a written informed consent form, approved by the local Ethical Committee. All procedures carried out herein complied with the principles laid down by the Declaration of Helsinki, revised Hong Kong 1989.

A preliminary screening procedure was carried out for identifying non-schizophrenia psychotic disorders, pseudo-TRS, non-TRS, and TRS patients. This procedure has been described elsewhere (6). For all patients, the following set of clinical-demographic data were recorded: age; gender; education years; age at disease onset (AaO); duration of illness (DoI); age at first psychiatric evaluation; history of substance, alcohol, or drug abuse; everyday living functional milestones (4). The following rating scales were administered by two experienced raters: the Clinical Global Impression-Severity (CGI-S); the Positive and Negative Syndrome Scale (PANSS); the Neurological Evaluation Scale (NES) (10); the Drug Attitude Inventory (11).

Patients were assessed for the following cognitive domains' performances: Sustained and Selective Attention by the Continuous Performance Task (CPT); Verbal Memory by the List Learning task; Visuospatial Memory (VSM) by the Brief Visuospatial Memory test-Revisited; Working Memory by the Digit Sequencing task; Verbal Fluency by the Category Instances task and the Controlled Oral Word Association test; Problem Solving by the Tower of London task; Speed of Information Processing by the Symbol Coding task. Raw data from each task were adjusted in corrected scores, according to values in the Italian normative population (12–14). High corrected scores corresponded to better preservation of cognitive status.

All statistical procedures were carried out by using the SPSS 24.0®. Descriptive statistics were used to report clinical and socio-demographic data. Independent-sample Student's *T*-test was used to compare quantitative data among diagnostic groups. In all tests, significance was set at *p* < 0.05 (two-tailed). Analysis of correlation was performed by Pearons's or Spearman's test, for continuous and categorical variables respectively. Multivariate linear regression analysis was used to perform both hierarchical linear regression (HLR) and mediation analyses.

## Results

### Group comparison

**Figure 1:**
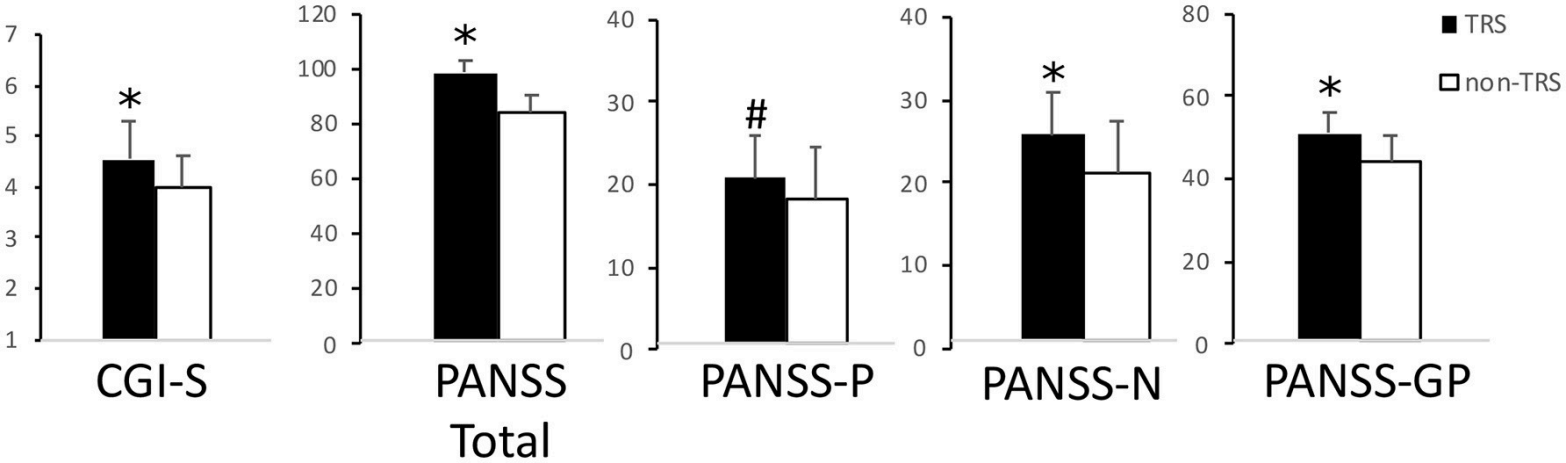
Disease severity and psychotic symptoms. In this picture are reported TRS and non-TRS groups' mean scores + standard deviations on the (from left to right): Clinical Global Impression-Severity (CGI-S) scale; Positive and Negative Syndrome Scale (PANSS) Total score; PANSS Positive Symptoms' Subscale (PANSS-P); PANSS Negative Symptoms' Subscale (PANSS-N); PANSS General Psychopathology Subscale (PANSS-GP). Note the different scales on multiple graphics. *p < 0.05 at the Student's *t*-test. ^#^Trend toward significance (*p* = 0.06).

A total of 73 schizophrenia patients enrolled in the study were subdivided in TRS (*n* = 41) and non-TRS (*n* = 32) ones. Age [*t*_(1, 71)_ = 1.66; *p* > 0.05], gender (χ = 1.64; *p* > 0.05), and education age [*t*_(1, 71)_ = 1.45; *p* > 0.05] were not significantly different between groups. Disease severity and psychotic symptoms were significantly more severe in TRS patients compared to non-TRS [Student's *t*-test; CGI-S: *t*_(1, 71)_ = 3.48; *p* = 0.001; PANSS Positive Score: *t*_(1, 71)_ = 1.92; *p* = 0.059; PANSS Negative Score: *t*_(1, 71)_ = 3.99; *p* < 0.0005; PANSS General Psychopathology (GP) Score: *t*_(1, 71)_ = 3.21; *p* = 0.002; PANSS Total Score: *t*_(1, 71)_ = 3.79; *p* < 0.0005] (Figure 1).

### Correlation analysis

In TRS patients, Pearson's test revealed significant positive correlations between disease severity and psychotic symptoms (PANSS Positive: *r* = 0.51, *p* = 0.001; PANSS Negative: *r* = 0.59, *p* < 0.0005; PANSS-GP: *r* = 0.58, *p* < 0.0005) or NSS (NES score: *r* = 0.44, *p* = 0.005), and inverse significant correlations between disease severity and verbal fluency performances (*r* = −0.35, *p* = 0.03) or VSM score (*r* = −0.33, *p* = 0.03).

In non-TRS patients, disease severity showed significant negative correlations with age (*r* = −0.38, *p* = 0.03) and duration of disease (*r* = −0.36, *p* = 0.04) and significant positive correlations with psychopathology (PANSS Positive: *r* = 0.50, *p* = 0.004; PANSS Negative: *r* = 0.41, *p* = 0.02; PANSS-GP: *r* = 0.56, *p* = 0.001), but not with NSS. Lifetime work occupation (ρ = −0.37, *p* = 0.03), residential status (ρ = −0.40; *p* = 0.02), and history of drug abuse (ρ = 0.42; *p* = 0.02) were also significantly correlated with disease severity at the Spearman's ρ test in these patients.

### Hierarchical linear regression

We used a hierarchical linear regression (HLR) approach to evaluate which variables explained the most part of variation in CGI-S score. PANSS Negative score was the variable that explained the most variance in CGI-S (Model 1: *F* = 21.22; *p* < 0.0005; *R*^2^ = 0.36; standardized β = 0.599). PANSS Positive score was the only other variable whose addition in the model led to a statistically significant increase in *R*^2^ (Model 2: *F* = 17.87; *p* < 0.0005; *R*^2^ = 0.49; standardized β PANSS Negative = 0.492; standardized β PANSS Positive = 0.380).

In non-TRS patients, the HLR approach showed that inclusion of PANSS-GP score explained substantial variation in CGI-S (Model 1: 13.64; *p* = 0.001; *R*^2^ = 0.313; standardized β = 0.559) and no other variable added significant variation to the equation.

### Mediation analysis

In order to make the relationships among these variables clearer, we performed a series of mediation analysis based on the Baron and Kenny four-step model (15). We started from the hypothesis that the variables responsible for the highest variance in HLR may mediate the relations with disease severity of the variables found associated to CGI-S in the correlation analysis.

According to correlation analysis, all variables included in the regression analysis were significant predictors of the outcome variable CGI-S (Step 1).

**Figure 2:**
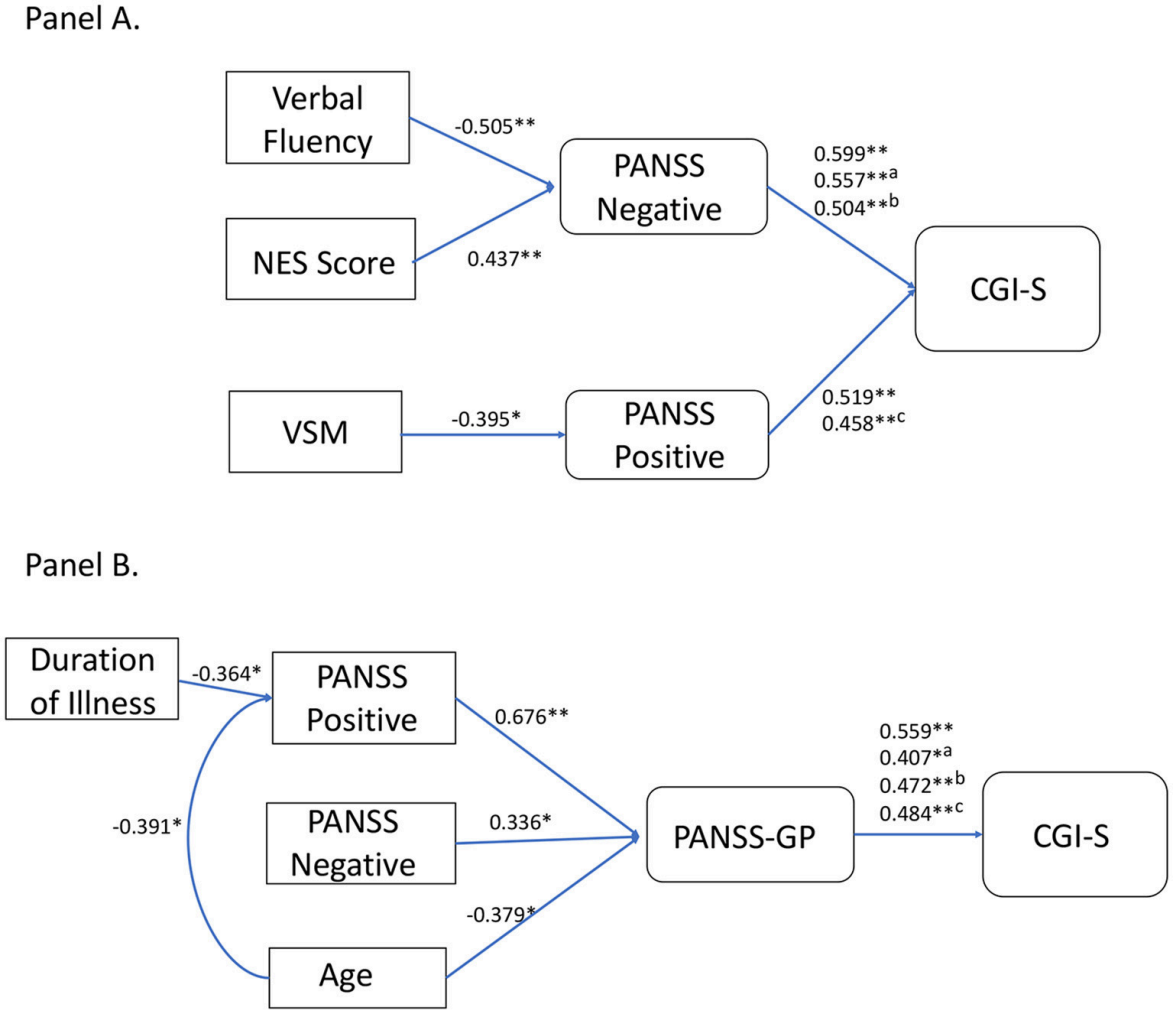
Graphical rendering of mediation analysis. Panel **(A)** reports outputs of mediation analysis for the TRS group. Causal variable is on the left and outcome variable on the right. Significantly associated variables are linked by connection lines. Above connection lines are reported standardized beta values from linear regression analyses, along with p values (*p < 0.05; **p < .005). For PANSS Negative connection with CGI-S, we reported uncontrolled, Verbal Fluency controlled (a), and NES controlled (b) standardized betas. Verbal Fluency and NES score were significantly predictive of CGI-S (standardized B = −0.34, *p* = 0.03; standardized B = 0.438, *p* = 0.005, respectively), but significance was lost after controlling for PANSS Negative (standardized B = −0.06, *p* > 0.05; standardized B = 0.218, *p* > 0.05). For PANSS Positive connection with CGI-s, we reported uncontrolled and VSM controlled (c) standardized betas. VSM was significantly predictive of CGI-S (standardized B = −0.334, *p* = 0.03), however significance was lost after controlling for PANSS Positive (standardized B = −0.153, *p* > 0.05). Panel **(B)** reports outputs of mediation analysis for the non-TRS group. For PANSS General Psychopathology (GP) connection with CGI-S, we reported uncontrolled, PANSS Positive controlled (a), PANSS Negative controlled (b), and age controlled (c) standardized betas. PANSS Positive, PANSS Negative, age, and duration of illness were significantly predictive of CGI-S (standardized B = 0.500, *p* = 0.004; standardized B = 0.417, *p* = 0.02; standardized B = −0.382, *p* = 0.03; standardized B = −0.362, *p* = 0.04, respectively), but significance was lost after controlling for PANSS-GP (standardized B = 0.225, *p* > 0.05; standardized B = 0.258, *p* > 0.05; standardized B = −0.198, *p* > 0.05, respectively) or PANSS Positive in the case of duration of illness (standardized B = −0.196, *p* > 0.05).

In TRS patients, the putative mediator variables were PANSS Negative score or PANSS Positive score. Verbal Fluency, NSS, and PANSS Positive score were significant predictors of the outcome variable PANSS Negative score (Step 2), while VSM score and PANSS-GP were not (Step 2 not met; analysis stopped). PANSS Negative score was significantly predictive of the outcome variable CGI-S when controlled for either Verbal Fluency, NSS, or PANSS Positive (Step 3). Verbal Fluency and NSS were no more significantly predictive of CGI-S score when controlled for PANSS Negative (Step 4), indicating that their relations with CGI-S may be partially mediated by negative symptoms. On the contrary, PANSS Positive was still significantly predictive of CGI-S when controlled for PANSS Negative, indicating that negative symptoms did not mediate the relation between positive symptoms and disease severity. VSM score, however, was significantly predictive of the outcome variable PANSS Positive (Step 2). PANSS Positive was predictive of CGI-S score after controlling for VSM score (Step 3), while VSM score was no more significantly predictive of CGI-S score after controlling for PANSS Positive (Step 4), thereby indicating that the relation between VSM and CGI-S was partially mediated by positive symptoms. Alternative models, using different combinations of causal and moderator variables, were investigated, however none of these yielded significant results (data not shown). The results of this analysis are illustrated in Figure 2A.

In non-TRS patients, mediation analysis showed that the most important mediator variable was PANSS-GP, which agreed with results of the HLR. Among the variables correlated with CGI-S, PANSS Positive, PANSS Negative, and age were significantly predictive of the outcome variable PANSS-GP (Step 2). PANSS Positive, PANSS Negative, and age were no more significantly predictive of CGI-S score when controlled for PANSS-GP (Step 3). PANSS-GP was still significantly predictive of CGI-S score after controlling for PANSS Positive, PANSS Negative, or age (Step 4). Alternative models were also investigated. The only other significant mediation model was found for PANSS Positive as a mediation variable for age and duration of illness effects on CGI-S. The results of this analysis are illustrated in Figure 2B.

## Discussion

The present work was aimed at dissecting some of the distinctive clinical features that affect disease severity in schizophrenia patients responsive to antipsychotic medications compared to TRS ones. We observed directional relationships among the variables accounted herein and disease severity, that were sharply divergent for TRS and non-TRS. Indeed, TRS has been considered a unique neurobiological clinical entity (16–18), with its proper pathophysiology, clinical presentation, and disease course (5, 7). The differences in clinical determinants of disease severity found in the present study comply with this view.

Notably, in TRS patients the most relevant clinical variable in determining disease severity was found to be the extent of negative symptoms. The impact of negative symptoms on disease severity does not appear attributable to their higher severity in TRS, since global psychotic symptoms as well as each psychotic symptom domain have been found more severe in TRS compared to non-TRS patients herein. Indeed, the association between negative symptoms and lack of response to antipsychotics had been classically reported (19, 20). Also, it has to be noted that, although being less severe than in TRS patients, the most relevant clinical variable in determining disease severity in non-TRS patients was PANSS General Psychopathology subscale score, which in turn accounts for the effects on disease severity of positive symptoms, negative symptoms, and duration of the illness. These elements let hypothesize a tight and putatively neurobiologically-determined connection between negative symptoms and TRS, affecting disease severity.

Relevance of negative symptoms on disease severity in TRS patients may lead to two alternative explanations: (i) patients with a larger extent of negative symptoms are considered to be TRS since these symptoms may not be impacted by antipsychotic agents; indeed, a large metanalysis of randomized placebo-controlled trials failed to find significant clinical effects of antipsychotics on negative symptoms (21); (ii) patients with a TRS suffer from a neurobiologically distinct form of the disease, which express symptomatically with prominent alterations in cognitive and negative symptoms. Indeed, there is strong evidence that cognitive dysfunctions are strictly interconnected with negative symptoms (22, 23).

The cross-sectional nature of this study does not allow to solve this issue. However, some clarifications may derive from mediation analysis. In TRS patients, mediation analysis showed that negative and positive symptoms directly and independently affected disease severity. Negative symptoms partially mediated the effects on disease severity of verbal fluency deficits and high-level neurological soft signs. Positive symptoms partially mediated the effects of visuospatial memory deficits. These data imply a strong distal effect of cognitive dysfunctions and neurological soft signs on psychopathology and disease severity in TRS patients. It has been proposed that cognitive deficits in schizophrenia may underlie proper and distinct neurobiology (24). Also, cognitive deficits and severe neurological soft signs may stem from more relevant neurodevelopmental aberrations in schizophrenia patients. Therefore, it should be hypothesized that TRS patients are a subset of schizophrenia patients whose relevant cognitive deficits and high-level neurological soft signs, of putative neurodevelopmental origin, in turn determine severe negative and positive symptoms, affecting disease severity. These theoretical causal inferences need to be demonstrated by means of *ad hoc* designed longitudinal designs.

Notably, determinants of disease severity are sharply divergent and do not involve neurological soft signs or cognitive alterations. Indeed, in non-TRS patients, general psychopathology partially mediated the effects of positive and negative symptoms, age, and duration of illness on disease severity. These results suggest that other clinical variables, not accounted herein, may have a major role in determining disease severity in non-TRS patients.

The results of this study should be interpreted in the light of its limitations: the sample size was relatively small, although TRS is a subpopulation of the whole schizophrenia patients and a representative sample is expected to be lower than that needed to study schizophrenia; rating scale scores may have been partially biased by antipsychotic treatment; selection of non-TRS patients was among patients initially suspected to be non-responsive to antipsychotic regimens and for this reason referred to our specialist unit, which may cause inclusion of severe, albeit non-TRS, patients and may mitigate differences with TRS patients.

## Author contributions

FI and AdB designed the study. CA, BA, AB, MM, LD, DN, and ER recruited the patients and administered assessment tools. FI carried out data analysis. FI and AdB wrote the manuscript. All authors read, corrected, and approved the manuscript in its final form.

### Conflict of interest statement

The authors declare that the research was conducted in the absence of any commercial or financial relationships that could be construed as a potential conflict of interest.
